# GP- and practice-related variation in ambulatory sensitive hospitalisations of older primary care patients

**DOI:** 10.1186/s12875-020-01285-9

**Published:** 2020-10-24

**Authors:** Leah Palapar, Laura Wilkinson-Meyers, Thomas Lumley, Ngaire Kerse

**Affiliations:** 1 Department of General Practice and Primary Health Care, School of Population Health, Faculty of Medical and Health Sciences, University of Auckland, Private Bag 92019, Auckland, 1142 New Zealand; 2 Health Systems Section, School of Population Health, Faculty of Medical and Health Sciences, University of Auckland, Auckland, New Zealand; 3 Department of Statistics, Faculty of Science, University of Auckland, Auckland, New Zealand

**Keywords:** Primary care practice variation, Physician variation, Hospitalisations, Older people

## Abstract

**Background:**

Reducing ambulatory sensitive hospitalisations (ASHs) is a strategy to control spending on hospital care and to improve quality of primary health care. This research investigated whether ASH rates in older people varied by GP and practice characteristics.

**Methods:**

We identified ASHs from the national dataset of hospital events for 3755 community-dwelling participants aged 75+ enrolled in a cluster randomised controlled trial involving 60 randomly selected general practices in three regions in New Zealand. Poisson mixed models of 36-month ASH rates were fitted for the entire sample, for complex participants, and non-complex participants. We examined variation in ASH rates according to GP- and practice-level characteristics after adjusting for patient-level predictors of ASH.

**Results:**

Lower rates of ASHs were observed in female GPs (IRR 0.83, CI 0.71 to 0.98). In non-complex participants, but not complex participants, practices in more deprived areas had lower ASH rates (4% lower per deprivation decile higher, IRR 0.96, CI 0.92 to 1.00), whereas main urban centre practices had higher rates (IRR 1.84, CI 1.15 to 2.96). Variance explained by these significant factors was small (0.4% of total variance for GP sex, 0.2% for deprivation, and 0.5% for area type). None of the modifiable practice-level characteristics such as home visiting and systematically contacting patients were significantly associated with ASH rates.

**Conclusions:**

Only a few GP and non-modifiable practice characteristics were associated with variation in ASH rates in 60 New Zealand practices interested in a trial about care of older people. Where there were significant associations, the contribution to overall variance was minimal. It also remains unclear whether lower ASH rates in older people represents underservicing or less overuse of hospital services, particularly for the relatively well patient attending practices in less central, more disadvantaged communities. Thus, reducing ASHs through primary care redesign for older people should be approached carefully.

**Trial registration:**

Australian and New Zealand Clinical Trials Register ACTRN12609000648224.

## Background

Hospital care accounts for 28% of health care expenditure [1], and there is increasing international attention to safely improve spending efficiency. One strategy is to focus on preventing admissions for conditions that are considered to be amenable to outpatient interventions, which are referred to as ambulatory sensitive hospitalisations (ASHs) in New Zealand. Comparing the magnitude of ASH across countries using available estimates can be difficult, as ASH prevalence can substantially vary according to the definition used (there are a number of ASH condition sets available in the literature) [2]. However, there is some consistency in countries continuing to report relatively stable or increasing ASH over time despite efforts geared towards reducing these potentially avoidable hospital stays [3–5].

High ASH rates are widely believed to be reflective of issues in access to high-quality primary care [6]. In support of this, European countries with stronger primary care structures and support for accessibility, coordination, and comprehensiveness have been shown to have lower hospitalisation rates for asthma, COPD, and diabetes after adjusting for disease prevalence and hospital bed supply [7]. In addition, areas with more primary care physicians are reported to have lower ASH rates [8, 9], whereas having more hospital beds is associated with higher ASH rates for a wide range of conditions [6]. These studies on geographic variation of ASHs may be useful for decisions at the national and regional levels, but may be less valuable in ascertaining specific aspects of primary care practice that can be targeted to reduce ASH rates.

In New Zealand, where the health system is mainly tax-funded and provides universal coverage for hospital care and a subsidised primary care system [10], ASHs are routinely reported. Primary care physicians (referred to as general practitioners or GPs) function as gatekeepers to secondary care services, and primary care patients are free to enrol with whichever practice they choose to attend [10]. Although certain patient characteristics are already known to be associates of higher ASH rates such as older age [4, 11–14], the presence of co-morbidities [4, 12, 13, 15, 16], and greater deprivation [4, 15], it is still unclear whether the risk of hospital admission varies according to the characteristics of GPs and practices. There is some evidence that age and sex of the GP and GP list size have a direct association with ASH rates [14, 17, 18]. Findings from reviews [9, 19] and cross-sectional studies [14, 17, 20] are inconsistent on the impact of practice characteristics such as practice size on hospitalisation rates [8]. One systematic review suggests that it is still unclear whether key organisational interventions or strategies such as hiring a practice nurse or offering disease management programmes influence ASH rates [9]. This article focuses on how characteristics of GPs and practices contribute to variation in rates of ASHs in older people.

## Methods

The present study is a secondary analysis of data from the Brief Risk Identification of Geriatric Health Tool (BRIGHT) trial, a cluster randomised controlled study that compared the effectiveness of a two-stage case-finding process in primary care against usual care in reducing residential care placement, hospitalisations, disability, and improving quality of life of older patients. The study ran from 2007 to 2012 and involved 3893 community-dwelling participants aged 75 years and over (65 and over for Māori) enrolled in 60 randomly selected general practices in three urban regions in New Zealand. Eligibility criteria and trial procedures have been described in detail elsewhere [21, 22]. In addition, all participating practices were surveyed to establish practice characteristics, and GP characteristics were ascertained from a survey sent to participating GPs [22, 23]. Some information was obtained from the New Zealand Medical Council database accessible to the public. For this analysis, we use data from 3755 of 3893 participants who completed baseline assessments, linked to GP- and practice-level data in determining GP- and practice-related variation in ASH rates with adjustment for an older person’s individual health and demographic characteristics.

Our variables of interest for this analysis are described in Tables 1 and 2. Based on the literature on medical practice variations [30–32], we selected 8 GP characteristics that may influence individual practice styles such as sex, training, and clinical experience. We also included 13 practice characteristics that may place opportunities or constraints on how GPs practice, such as age and ethnic composition of the practice population, number of GPs in the practice, level of deprivation and area type (main urban centre versus other types, which include satellite and independent urban communities) of the practice location, and performance of five activities to promote early problem detection in older patients (using assessment tools, auditing the practice, having clinics for frail older patients, home visiting, and systematically contacting patients proactively).

**Table 1:** Characteristics of participants in the entire sample (*n* = 3755), complex (*n* = 1374) and non-complex subgroups (*n* = 2241)

Variable	Missing	Entire sample	Complex subgroup	Non-complex subgroup	Notes
		(*n* = 3755)	(*n* = 1374)	(*n* = 2241)	
		Freq (%)	Freq (%)	Freq (%)	
Age, mean (SD)	1	79.8 (4.6)	80.1 (4.6)	79.6 (4.6)	
Sex	0				
Male		1693 (45.1)	693 (50.4)	947 (42.3)	
Female		2062 (54.9)	681 (49.6)	1294 (57.7)	
Ethnicity	2				Other ethnicity includes Pacific, Asian, and African
New Zealand Maori		177 (4.7)	54 (3.9)	115 (5.1)	
New Zealand European		2817 (75.1)	1058 (77.0)	1666 (74.4)	
European		645 (17.2)	222 (16.2)	390 (17.4)	
Other		114 (3.0)	40 (2.9)	68 (3.0)	
Marital status	39				
Married		1985 (53.4)	743 (54.4)	1183 (53.4)	
Widow or widower		1417 (38.1)	497 (36.4)	863 (38.9)	
Single or divorced		314 (8.5)	126 (9.2)	171 (7.7)	
Living arrangement	12				
Alone		1550 (41.4)	541 (39.4)	941 (42.1)	
With only spouse or partner		1993 (53.3)	740 (53.9)	1194 (53.4)	
Other		200 (5.3)	91 (6.6)	101 (4.5)	
NZDep06 of home address	150				Higher decile areas in the 2006 New Zealand Index of Deprivation (NZDep06) [24] represent areas with greater levels of deprivation
1st-2nd decile (low deprivation)		1012 (28.1)	318 (24.3)	658 (30.4)	
3rd-5th decile		1256 (34.8)	465 (35.5)	748 (34.6)	
6th–10th decile (high deprivation)		1337 (37.1)	526 (40.2)	759 (35.1)	
Education	154				
Completed primary		522 (14.5)	214 (15.8)	302 (13.7)	
Completed secondary		1645 (45.7)	625 (46.2)	999 (45.3)	
Completed tertiary		1434 (39.8)	515 (38.0)	903 (41.0)	
5 or more health problems	140	312 (8.6)	312 (22.7)	0 (0.0)	Summary score adding the number of positive responses reported by participants to 14 health conditions: hypertension, asthma, diabetes, arthritis, epilepsy, Parkinson’s disease, osteoporosis, myocardial infarction or angina, stroke, chronic lung problems, hip fracture, knee replacement, hip replacement, depression or mental illness
3 or more medication types	20	2267 (60.7)	1134 (82.8)	1054 (47.2)	Participant-reported number of medication types
AMTS score ≤ 6	33	52 (1.4)	21 (1.5)	28 (1.3)	A score of 6 or less in the Abbreviated Mental Test Score (AMTS) [25] is indicative of cognitive impairment
GDS-15 score ≥ 5	24	342 (9.2)	188 (13.7)	136 (6.1)	A score of 5 or more in the 15-item Geriatric Depression Scale (GDS-15) [26] is suggestive of depression
Social support score, mean (SD)	340	28.4 (3.1)	28.0 (3.2)	28.7 (3.0)	Higher scores in the 11-item Duke Social Support Index [27] represent more social interaction and support
Inadequate physical activity	229	1711 (48.5)	748 (55.9)	954 (44.0)	Exercises less than 30 min/5 times per week considered inadequate
Current or ever smoked	5	2078 (55.4)	849 (61.8)	1147 (51.2)	
Alcohol consumption	145	2197 (60.9)	811 (59.4)	1373 (61.7)	
Frequency of alcohol intake	162				
Daily or almost daily		1177 (32.8)	430 (31.6)	741 (33.5)	
Weekly		532 (14.8)	198 (14.6)	331 (15.0)	
Monthly		469 (13.1)	178 (13.1)	287 (13.0)	
Never		1415 (39.4)	554 (40.7)	854 (38.6)	
High nutritional risk	25	1199 (32.1)	610 (44.6)	573 (25.7)	A score of 6 or more in the Australian Nutrition Screening Initiative [28] is suggestive of high nutritional risk
No ASH 18 months prior	7	3258 (86.9)	1090 (79.5)	2048 (91.5)	
Non-overnight ASH 18 months prior	7	79 (2.1)	39 (2.8)	35 (1.6)	

*Freq* frequency, *SD* standard deviation, *NZDep06–2006* New Zealand Index of Deprivation, *AMTS* Abbreviated Mental Test Score, *GDS-15* 15-item Geriatric Depression Scale

**Table 2:** Characteristics of sample GPs (*n* = 125) and practices (*n* = 60)

Variable	Missing	Freq (%)	Notes
GP characteristics (*n* = 125)			
Sex	0		
Male		54 (43.2)	
Female		71 (56.8)	
Country trained	2		Overseas-trained GPs include those trained in the UK, South Africa, Sri Lanka, among others
New Zealand		83 (67.5)	
Overseas		40 (32.5)	
Years since graduation, mean (SD)	2	24.3 (9.0)	
Years in general practice, mean (SD)	16	17.4 (8.7)	
Years at this practice, mean (SD)	16	12.8 (9.2)	
Number of older patients, mean (SD)	0	65.6 (57.7)	
0.6 full time equivalent or higher	16	84 (77.1)	Full time equivalent calculated as number of clinical sessions per week / 10
Position	17		Owners refer to sole owners or partners, associates are GPs on the practice partnership track, and locums are GPs who are not owners or salaried employees of the practice
Owner or associate		93 (86.1)	
Locum or employed GP		15 (13.9)	
Practice characteristics (*n* = 60)			
NZDep06 of practice location	0		Higher decile areas represent areas with greater levels of deprivation
1st to 8th decile		43 (71.7)	
9th to 10th decile		17 (28.3)	
Area type of practice location	0		Determined using geographic concordance files from Statistics New Zealand [29]; other area types include satellite urban communities and independent urban communities
Main urban centre		56 (93.3)	
Other		4 (6.7)	
≥ 10% patients aged 75+	11	16 (32.7)	
≥ 10% Maori patients	11	18 (36.7)	
5000 enrolled patients or more	11	19 (38.8)	
7 GPs or more	6	19 (35.2)	
≥ 30% locum GPs	6	18 (33.3)	We assumed that having a smaller proportion of locum GPs promotes continuity of care
Formal assessment tool	3	4 (7.0)	Always using a formal assessment tool to help determine whether older patients have special needs
Clinical audit for frail older patients	3	7 (12.3)	Regularly auditing the practice to identify frail older people who may need additional support or an assessment
Clinics for frail older patients	4	21 (37.5)	Regularly having clinics for frail older patients to identify need or disability risk
Home visits	3	46 (80.7)	Providing regular home visits for older patients who need them
Proactive contacts, any type	3	45 (79.0)	Systematically contacting patients for any of the three reasons specified
Missed appointments	3	43 (75.4)	
Prescriptions not renewed	4	15 (26.8)	
No check up in a long time	4	21 (37.5)	
Number of practice activities	3		A summary score adding the number of positive responses reported by practices to the five proactive processes described above (using assessment tools, auditing the practice, having clinics for frail older patients, home visiting, and systematically contacting patients); an alternative score that considered types of proactive contacts as separate activities (range 0–7) was also calculated
None		4 (7.0)	
1 to 2		36 (63.2)	
3 to 5		17 (29.8)	

*Freq* frequency, *SD* standard deviation, *NZDep06–2006* New Zealand Index of Deprivation

Hospital admissions 18 months prior to baseline and up to 36 months from baseline were determined by matching participant’s National Health Index (NHI) numbers to records in the National Minimum Dataset (NMDS) – a collection of information on hospital events in New Zealand. We used ICD-10 codes to identify admissions for a nationally defined [33] set of conditions considered to be sensitive to ambulatory care (Appendix) and applied the current standardised formula [34] to calculate the total number of urgent and semi-urgent ASH events (referred to in the NMDS as acute and arranged admissions, respectively) [35], including non-overnight stays (events with a length of stay of zero) but excluding non-urgent (waiting list) admissions.

Stata 11.0 and R were used to perform Poisson mixed modelling with ASHs over 36 months as the dependent variable. We fitted random intercepts models (level 1 participants, level 2 GPs, level 3 practices) that included a parameter to account for differences in the length of time participants were exposed to the risk of admission – as in participants who were unable to complete the trial due to poor health, residential care placement, or death. Our modelling procedure was as follows:

1. Specified a base model with two predictors: group assignment in the BRIGHT trial and number of ASH events in the 18-month period prior to baseline;
2. Separately added 17 candidate participant-level predictors of ASH (Table 1) to the base model, and where *p* ≤ 0.05, the variable advanced to the next analysis step;
3. Specified a *full base model* by simultaneously adding participant-level predictors of ASH to the base model as determined in the previous analysis step; and
4. Separately added the 8 GP-level variables and 13 practice-level variables of interest.

The final step yields an estimate of the adjusted variation in enrolled older participants’ 36-month ASH rate for a particular GP and practice characteristic. Estimates are reported as incident rate ratios with interval estimates obtained to a 95% level of confidence.

We hypothesised that the number of ASH events and its predictors would differ based on the type of care likely to be needed by older people. Participants who have been diagnosed with five or more conditions, had myocardial infarction or angina, stroke, chronic bronchitis, emphysema, or chronic lung problems due to cigarette smoking were considered to have complex care needs as these participants are more likely to need personal care, household support, or community-based rehabilitation services; otherwise, participants were categorised as non-complex. Three sets of the models described above were fitted: for the entire sample; for the complex participant subgroup; and for the non-complex participant subgroup.

We performed sensitivity analyses to examine the adjusted variation in 36-month ASH rates when we:

1. Fitted negative binomial models to relax the distributional assumption of equidispersion (i.e., variance is equal to the mean) [36] in Poisson models;
2. Restricted our definition of ASHs by excluding admissions having a length of stay of zero (non-overnight stays) following Ministry of Health [37] recommendations due to inconsistent reporting of short stay Emergency Department events prior to 2012;
3. Accounted for regional secondary care supply by adding practices’ District Health Board (DHB, regional funding body) in the model, as availability of hospital beds is a recognized driver of high ASH rates [6]; and
4. Pooled the estimates from five imputations for missing participant characteristics.

For associations related to complexity, we additionally investigated variation in ASH rates when four alternative thresholds for complexity were used. We varied the definition of complexity by reducing the comorbidity level cut-off to (1) four health conditions or (2) three health conditions and by (3) considering low levels of social support in combination with or (4) in addition to comorbidity level and types of health conditions.

## Results

Tables 1 and 2 presents the characteristics of 3755 BRIGHT participants who completed baseline assessments and their 125 GPs in 60 participating practices, of which 1374 (38.0%) satisfied at least one of the criteria for having complex care needs. There were slightly more female (56.8%) than male GPs. Only 4 of 60 practices were located in areas other than main urban centres e.g., satellite or independent urban communities (6.7%); 17 (28.3%) were in areas of high deprivation (NZDep deciles 9–10). Most practices routinely performed at least one of the five activities that can help identify frail older participants who need assistance (93.0%).

In the 18-month period prior to baseline assessment, the majority of the sample was not hospitalised for ASH conditions (86.9%). Only 2.1% of participants had a non-overnight stay for an ASH condition. ASH rates from baseline to 36-month follow-up in complex participants were significantly higher than non-complex participants in the unadjusted model (387 and 187 admissions per 1000 older person years, respectively; IRR 2.08, CI 1.93 to 2.24). Participant-level variables included in the full base models, which are shown in Table 3, accounted for 13.4% of the variation in 36-month ASH rates in the entire sample, 14.7% in the complex subsample, and 8.7% in the non-complex subsample.

**Table 3:** Full base models^a^ of 36-month ASH rates for entire sample, complex and non-complex subgroups

Variables	Entire sample		Complex subgroup		Non-complex subgroup	
	(*n* = 3755)		(*n* = 1374)		(*n* = 2241)	
	IRR	95% CI	IRR	95% CI	IRR	95% CI
BRIGHT intervention group	1.07	0.88–1.30	1.04	0.82–1.32	1.18	0.92–1.51
ASHs 18 months prior to baseline	1.26	1.23–1.28	1.24	1.21–1.27	1.30	1.23–1.39
Age	1.06	1.05–1.06	1.04	1.03–1.05	1.06	1.05–1.08
Sex						
Male (reference)						
Female^a^						
Ethnicity						
NZ European (reference)						
NZ Maori	0.84	0.70–1.01	1.03	0.80–1.33		
Other ^b^	0.76	0.68–0.84	0.65	0.55–0.75		
Marital status						
Married (reference)						
Widow or widower	1.32	1.22–1.43	1.32	1.18–1.47	1.32	1.17–1.48
Single or divorced	1.15	1.00–1.32	1.11	0.91–1.34	1.03	0.82–1.30
Living arrangement						
Alone (reference)						
With only spouse or partner	0.76	0.70–0.82	0.78	0.70–0.87	0.76	0.67–0.85
Other	1.06	0.91–1.23	1.04	0.85–1.27	1.01	0.79–1.30
NZDep06 ^c^ of home address	1.04	1.03–1.06	1.03	1.01–1.06	1.05	1.02–1.07
Education						
Completed primary (reference)						
Completed secondary	0.82	0.74–0.91	0.86	0.74–0.99	0.82	0.70–0.96
Completed tertiary	0.69	0.62–0.77	0.82	0.70–0.96	0.60	0.50–0.71
Number of health problems	1.27	1.24–1.30	1.16	1.12–1.20	1.34	1.28–1.41
Number of medication types	1.12	1.10–1.13	1.08	1.06–1.10	1.13	1.10–1.15
AMTS score ^d^	0.91	0.88–0.95			0.88	0.83–0.93
GDS-15 score ^e^	1.10	1.09–1.12	1.07	1.04–1.09	1.13	1.10–1.16
Social support score ^f^	0.97	0.96–0.98	0.98	0.96–1.00	0.98	0.96–0.99
Adequate physical activity	0.68	0.63–0.73	0.69	0.61–0.77	0.75	0.66–0.84
Does not smoke					1.13	1.01–1.27
Does not drink	1.24	1.15–1.34	1.19	1.07–1.32	1.32	1.18–1.49
Frequency of alcohol intake						
Daily or almost daily (reference)						
Weekly	1.02	0.89–1.15	1.07	0.90–1.27	0.97	0.79–1.18
Monthly	1.25	1.10–1.41	1.01	0.85–1.21	1.55	1.30–1.86
Never	1.32	1.20–1.45	1.22	1.07–1.38	1.48	1.28–1.70
Nutritional risk score ^g^	1.09	1.08–1.10	1.04	1.02–1.06	1.10	1.08–1.12

^a^ Candidate participant characteristics not included in fully adjusted analysis are represented as white space; ^b^ Includes European, Pacific, Asian, and African; ^c^ 2006 New Zealand Index of Deprivation [24]; ^d^ Abbreviated Mental Test Score [25]; ^e^ 15-item Geriatric Depression Scale [26]; ^f^ measured using the 11-item Duke Social Support Index [27]; ^g^ measured using the Australian Nutrition Screening Initiative [28]

**Fig. 1:**
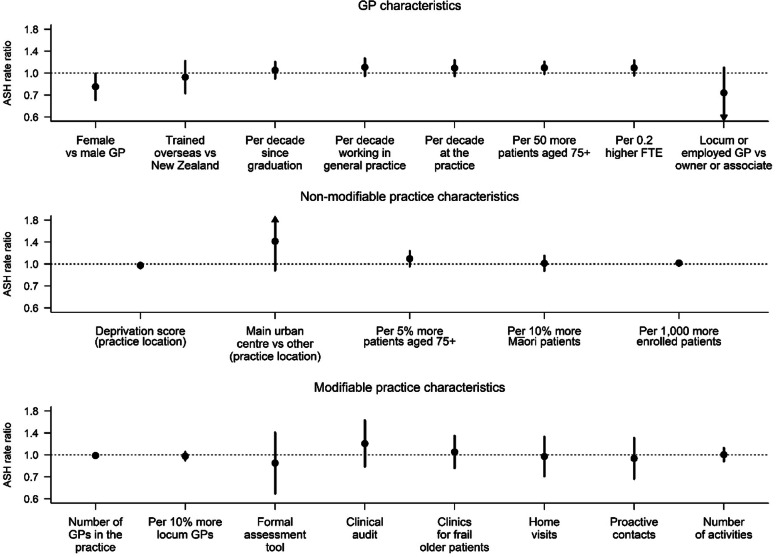
Variation in adjusted 36-month ASH rates according to GP and practice characteristics, **entire sample**^a, b^. ^a^ GP- and practice-level variables separately added to the full base model for the **entire sample** that includes group assignment in the BRIGHT trial, number of ASH events in the 18-month period prior to baseline, age, ethnicity, marital status, living arrangement, deprivation decile of participant’s home address, education, number of health problems, number of medications, Abbreviated Mental Test Score (cognition), Geriatric Depression Scale (GDS-15) score (depression), social support score, physical activity, alcohol consumption, frequency of alcohol intake, and nutritional risk score. ^b^ Estimates < 0.6 and > 1.8 are marked as ▼and ▲, respectively

Variation in 36-month ASH rates after fully adjusting for participant characteristics, determined by separately adding GP characteristics, non-modifiable practice characteristics, and modifiable practice characteristics to the full base model, are summarised in Fig. 1. ASH rates were significantly lower by 17% in participants attending female GPs (IRR 0.83, CI 0.71 to 0.98). Qualitatively similar variation according to GP sex were obtained when we (1) fitted a negative binomial rather than Poisson model (IRR 0.81, CI 0.68 to 0.96); (2) restricted our definition of ASHs by excluding day cases (IRR 0.83, CI 0.71 to 0.98); (3) added practices’ DHB to the model as a proxy of regional secondary care supply (IRR 0.84, CI 0.70 to 1.00); and (4) pooled the estimates from multiply-imputed participant characteristics (pooled IRR 0.84, CI 0.71 to 0.99). Adding GP sex to the model increased the proportion of variance explained from 13.4% (full base model) to 13.8%.

There were no other significant associations between GP and practice characteristics and ASH rates.

### Subgroup analyses

#### Complex participants

There was no significant variation in fully adjusted ASH rates by GP and practice characteristics when the analysis was restricted to participants with complex care needs.

#### Non-complex participants

**Fig. 2:**
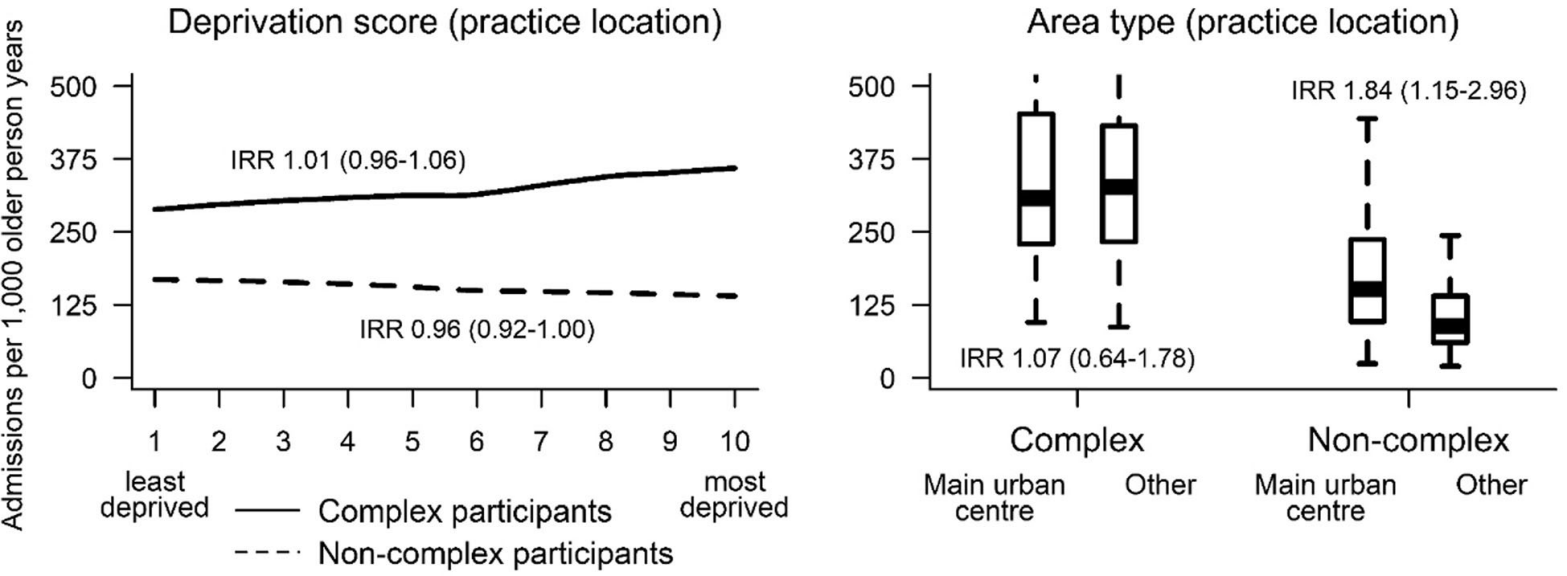
Adjusted ASH per 1000 person-years^a^ by practice deprivation and area type, **complex**^b^ **or non-complex**^c^ **subgroups.** ^a^ Aged 75+ in primary care. ^b^ Practice-level variables separately added to the full base model for the **subgroup of participants with complex care needs** that includes group assignment in the BRIGHT trial, number of ASH events in the 18-month period prior to baseline, age, ethnicity, marital status, living arrangement, deprivation decile of participant’s home address, education, number of health problems, number of medications, Geriatric Depression Scale (GDS-15) score (depression), social support score, physical activity, alcohol consumption, frequency of alcohol intake, and nutritional risk score. ^c^ Practice-level variables separately added to the full base model for the **subgroup of non-complex participants** that includes group assignment in the BRIGHT trial, number of ASH events in the 18-month period prior to baseline, age, marital status, living arrangement, deprivation decile of participant’s home address, education, number of health problems, number of medications, Abbreviated Mental Test Score (cognition), Geriatric Depression Scale (GDS-15) score (depression), social support score, physical activity, smoking, alcohol consumption, frequency of alcohol intake, and nutritional risk score

**Fig. 3:**
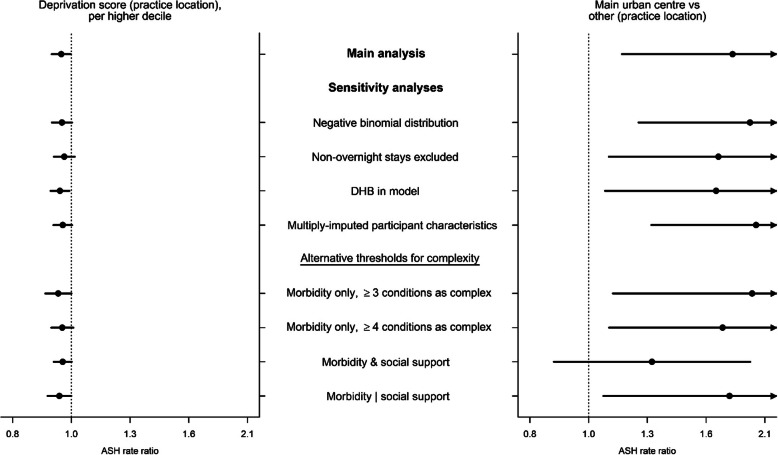
Sensitivity analyses for variation in ASH by practice deprivation and area type, **non-complex subgroup**^a^. ^a^ Deprivation decile and area type of practice location separately added to the full base model for the **subgroup of non-complex participants** that includes group assignment in the BRIGHT trial, number of ASH events in the 18-month period prior to baseline, age, marital status, living arrangement, deprivation decile of participant’s home address, education, number of health problems, number of medications, Abbreviated Mental Test Score (cognition), Geriatric Depression Scale (GDS-15) score (depression), social support score, physical activity, smoking, alcohol consumption, frequency of alcohol intake, and nutritional risk score

In fully adjusted analyses for the subgroup of participants with non-complex care needs, there were no GP characteristics and 2 of 13 practice characteristics significantly related to variation in ASH rates. Figure 2 shows that in non-complex participants, we observed a 4% lower rate of ASHs corresponding to each higher decile of deprivation of the practice location (IRR 0.96, CI 0.92 to 1.00) and an 84% higher rate in those attending practices in main urban centres compared to other areas (IRR 1.84, CI 1.15 to 2.96). Non-complex participants’ variation in ASH rates according to deprivation decile and area type of the practice location were qualitatively similar in sensitivity analyses (Fig. 3). The proportion of variance explained increased from 8.7% (full base model) to 8.9% in the practice deprivation model and 9.2% in the practice area type model.

## Discussion

The present study investigated whether rates of ASHs in older people varied over three years of follow-up according to the characteristics of their GPs and practices using data from participants and practices enrolled in a trial about care of older people. We found lower ASH rates in participants seen by female GPs, in non-complex participants attending practices located in areas of greater deprivation, and in non-complex participants attending practices located in areas other than main urban centres. There were very few significant GP- and practice-related variations in rates of ASHs in comparison to the number of factors we examined, and the amount of variation explained by the GP and practice characteristics was relatively small.

We found that participants seen by female GPs had lower rates of ASH. It is possible that female physicians, who are known to engage in more affective and partnership-building communication [38], interact with their patients in a more person-centred manner thus leading to trust, improved self-efficacy, and increased motivation to adhere to one’s treatment plan, which may contribute to better health outcomes [39]. Significantly fewer hospitalisations have also been previously reported in patients who experienced more person-centred physician-patient interactions (determined through direct observation) over a one-year period [40].

Increased deprivation is usually associated with higher health care utilisation including ASHs [4, 15]. Consistent with this, we found that if the *participant’s address* was in an area of greater deprivation, they had higher ASH rates, which may represent socioeconomic inequity. However, when we accounted for deprivation in the participant’s area of residence, multimorbidity, and other participant-level predictors of ASH, lower ASH rates were observed in non-complex participants if the *practice address* was in an area of greater deprivation. In this sample of urban practices (i.e., a few were located in small towns), we also found higher ASH rates in non-complex participants of practices *within* main urban centres (where the hospitals are) compared to those *further away* from the city centre. This finding may be related to the positive association between proximity to health care facilities and health care use, which is referred to as the distance decay effect [41]. Previous ASH studies examining the impact of different location types (e.g., ranging most urban to most rural) rather than a dichotomy report broadly similar associations – ASH rates are highest at both ends of the spectrum [13, 42]. Our findings may suggest over servicing in practices located in less deprived areas and within city centres, and appropriate care in the community in practices located in more deprived areas and those further away from city centres. It is possible that those who choose to practice in less central, socioeconomically disadvantaged areas tend to have a better understanding of their relatively high-needs, underserved practice populations [43] including socially patterned behaviour towards health and illness [44] and the economic burden of hospital care such as cost of travel to hospital or loss of income by the family caregiver [45]. However, as significant differences were only observed in non-complex participants, potentially unreported needs in participants who are sufficiently healthy to recover from an acute episode of illness is another plausible explanation. Without further information on participants’ functional ability, quality of life, or satisfaction with care, these observations in non-complex participants are perplexing – more admissions may be necessary if needs are not met, but overuse of hospital services should be avoided.

Overall, significant variation in rates of ASHs were observed in only a few of the 21 characteristics we examined: sex of the GP the older person sees, practice location, and neighbourhood deprivation of the practice they are enrolled in rather than modifiable aspects of primary care. Furthermore, the amount of variation explained by these significant factors was relatively small – the largest being 0.5% of the total variance. Taken together, another important interpretation is that a substantial proportion of ASH admissions in the older population may be difficult to prevent through structural modifications in primary care. Frequency and level of engagement with primary care may already be high at this age, especially for those who have complex care needs, thus attending a practice with proactive processes in place may no longer have a considerable impact on outcomes. Other studies have also shown that interpractice variability in ASH rates tends to be smaller at older ages [46]. This suggests that we may need to carefully approach primary care redesign for older people as we seek to reduce hospital overuse.

The present study linked administrative data on hospital events to patient-, GP-, and practice-level data from a large primary care-based trial, which made it possible to estimate variation related to GP and practice characteristics with adjustment for individual participants’ health and sociodemographic characteristics. Our results should be interpreted with caution considering that ASH rates varied significantly albeit minimally in only a few of the many potential primary care predictors examined – it is possible that these are due to chance alone, but it is reassuring that we also noted variation in the same direction in our sensitivity analyses. As this is an observational study that made use of available trial data, we are unable to establish causal relationships. The patients described in the present study were recruited from 60 practices in 3 DHBs with response rates of 47 and 52% at the patient and practice levels [22]; generalisability of findings may be limited to the group of practices interested in a trial about care of older people and to the types of patients who chose to participate. In addition, New Zealand has a publicly-funded acute hospital system which is therefore accessible. It will be interesting to compare associations in other country contexts as our observations may be different in other health systems.

Future investigations should examine hospital length of stay as an outcome as patients may have had more frequent but shorter admissions at the earlier course of their disease. Differentiating overnight from non-overnight admissions, ED presentation type (self- or GP-referred to hospital), and person-centred outcomes such as functional ability and quality of life may help ascertain hospital overuse.

## Conclusions

We found significant variation in ASH rates, which were related to the sex of the GP that older people see, and the location and neighbourhood deprivation of the practice they enrol in rather than modifiable aspects of primary care. It remains unclear whether lower ASH rates represent appropriate community-based care or unmet need for hospitalisation, particularly in relatively well older people. Given the push to reduce hospital overuse in many countries, general practice should lead the call to approach primary care redesign for older people carefully.

## Data Availability

Hospital data was requested from the Ministry of Health, and GP data was obtained from the publicly accessible New Zealand Medical Council database. BRIGHT trial datasets used in the present study are not publicly available as we did not seek participant request for this.

## Appendix

**Table 4:** ICD-10 codes for conditions considered to be sensitive to ambulatory care, NZ Ministry of Health [33]

Ambulatory sensitive conditions	Principal diagnosis codes
Angina and chest pain	I20, R072-R074
Asthma	J45, J46
Cellulitis	H000, H010, J340, L01-L04, L08, L980
Cervical cancer	C53
Congestive heart failure	I50, J81
Constipation	K590
Dental conditions	K02, K04, K05
Dermatitis and eczema	L20-L30
Diabetes	E10-E14, E162
Epilepsy	G40, G41, O15, R560, R568
Gastroenteritis/Dehydration	A02-A09, K529, R11
Gastroesophageal reflux disease	K21
Hypertensive disease	I10-I13, I15, I674
Kidney/Urinary infection	N10, N12, N136, N309, N390
Myocardial infarction	I21-I23, I241
Nutrition deficiency and anaemia	D50-D53, E40-E46, E50-E56, E58-E61, E63, E64, M833
Other ischaemic heart disease	I240, I248, I249, I25
Peptic ulcer	K25-K28
Respiratory infections – Pneumonia	J13-J16, J18
Rheumatic fever/Heart disease	I00-I02, I05-I09
Sexually transmitted infections	A50-A59, A60, A63, A64, I980, M023, M031, M730, M731, N290, N341
Stroke	I61, I63-I66
Upper respiratory tract and ear, nose and throat infections	J00-J04, J06, H65-H67

## Abbreviations

ASH: Ambulatory sensitive hospitalisation
BRIGHT: Brief Risk Identification of Geriatric Health Tool
DHB: District Health Board
GP: General practitioner
NHI: National Health Index
NMDS: National Minimum Dataset
